# A Mixed Methods Study of Ethnic Identity and Mental Health Recovery Processes in Minoritized Young Adults

**DOI:** 10.3390/healthcare12202063

**Published:** 2024-10-17

**Authors:** Kiara L. Moore, Aaron H. Rodwin, Rei Shimizu, Michelle R. Munson

**Affiliations:** 1 Silver School of Social Work, New York University, New York, NY 10003, USA; 2 School of Social Work, University of Alaska, Anchorage, AK 99508, USA

**Keywords:** mental health, cross-cultural, personal recovery, ethnic identity, diversity, young adult

## Abstract

Background/Objectives: Ethnic identity development is associated with positive mental health in young adults from ethnic minority groups. How a sense of belonging and attachment to one’s ethnic culture is related to personal mental health recovery remains unexplained. This study examines the experiences of ethnic minority young adults in the U.S. to understand the aspects of culture and identity development that are relevant to their recovery processes. Methods: Young adults who were living with chronic mental disorders were recruited from four rehabilitation programs. Interviews produced quantitative and qualitative data. An explanatory sequential mixed methods design was used to integrate the qualitative findings from a sub-group of young adults (*n* = 44) with the results from the quantitative study. Directed content analysis was used to analyze the qualitative data, and the integrated data were analyzed in joint displays. Results: The prominent themes characterizing ethnic identity development in personal recovery were (a) cultural history, traditions, and values; (b) mental illness stigma within the ethnic community; and (c) bias and discrimination in mental health services. Young adults with high ethnic identity development reported having more support from family, but they also described experiences with stigma and racism. Conclusions: The integrated results suggest that ethnic identity development promotes mental health recovery in minoritized young adults through social support and improved well-being and resilience. Experiences of intersectional stigma and structural racism associated with ethnic identity can interfere with self-determination and access to care among minoritized Hispanic/Latine, Black, and multiracial young adults in the U.S.

## 1. Introduction

Many chronic mental health problems first appear during early adulthood (ages 18–29) [1], a period when individuals are concerned with establishing their social identities [2]. Living with a mental health condition, such as a chronic mood or psychotic disorder, can interfere with developing a positive, coherent sense of self [3], and it can restrict exploring who to become and what to do with one’s life [4]. Distress and disability from symptoms, along with the effects of mental illness stigma, can disrupt progress toward critical milestones of adulthood, such as attaining education and occupational goals, fulfilling adult social roles and relationships, and gaining greater self-determination [5,6]. These interruptions may be more detrimental for young adults from minoritized ethnic groups that experience social disadvantages due to bias and discrimination [7,8]. Experiencing a mental illness during early adulthood increases the risk of persistent health inequalities and diminished recovery for ethnic minority adults [9,10].

Ethnic identity formation may play a role in recovery in minoritized young adults who are living with mental disorders. Late adolescence and early adulthood are thought to be stages when individuals focus more intently on exploring and establishing their ethnic identities [11,12]. Research on psychosocial development in early adulthood has typically shown that having a stable, affirmative sense of one’s ethnic identity is associated with positive mental health and psychological well-being in young adults from minoritized groups [11,13]. Ethnic identity development has also been indicated as a protective factor for minoritized young adults facing discrimination and adversity [13,14,15]. Moreover, substantial research on the concept of subjective, personal recovery has highlighted how a person’s ethnic culture and sense of belonging and involvement with their ethnic community are key factors in recovering from mental illness [16,17]. Mental health services that emphasize personal recovery show evidence of improving functioning and well-being among young adults with mental disorders [18,19]. However, ethnic and racial minority young adults continue to experience disparities in treatment [7,20,21]. Understanding the significance of ethnic identity could inform improvements to mental health services for young adults [22]. Yet, few studies have examined ethnic identity in connection with mental health recovery in minority young adults [21], and very few have considered the self-defined concept of personal recovery [5].

This study examines data from a sequential mixed methods research project [23] to address this gap in the literature. As a follow-up to our first quantitative study component, which assessed the association of ethnic identity development with personal recovery among a sample of Black and Hispanic/Latine young adults living with mental disorders in the U.S. (*n* = 83) [24], we analyze qualitative interviews conducted with a sub-group of the participants. The results from our first study component indicated that higher levels of ethnic identity development significantly predicted personal recovery (*b* = 0.74, *SE* = 0.30, *p* = 0.023), when controlling for age, gender, severity of symptoms, and ethnic–racial group in a multivariate regression model (*F* = 5.67, *p* < 0.001). To further explore these results in the present study, we analyze participants’ interviews for connections between ethnic identity and recovery processes and use joint displays to compare the qualitative data between participants with higher and lower levels of ethnic identity development. The qualitative findings of the present study provide context to our previous quantitative results and expand our understanding of the ethnic identity and personal recovery processes of minoritized young adults.

### 1.1. Ethnic Identity and Mental Health

Ethnic identity is a domain of one’s sense of self in terms of a specific ethnic group, typically inclusive of race, culture, language, and kinship [25]. Ethnic identity includes one’s sense of belonging and attachment to their group; perceptions, behaviors, and feelings due to group membership; and involvement in social and cultural practices of the group [12,25,26]. Developing one’s ethnic identity during early adulthood occurs through pursuing more knowledge of and participation in one’s ethnic community and cultural practices, and feeling a strong sense of belonging to and gratification in one’s group [11,27]. In addition to generally supporting mental health and well-being, a positive sense of ethnic identity can help young adults from minoritized groups to derive self-esteem, psychological adjustment, and social support from their ethnic communities [11,28]. These assets may help them to resist internalizing negative stereotypes and promote resilience when facing setbacks in life, including stigma and discrimination [29,30]. Studies have also found that ethnic identity development is associated with fewer mental illness symptoms among ethnic and racial minority young adults [14,31,32], suggesting it has protective effects in the context of clinical recovery [33]. Recently, ethnic identity has been included as a focus of clinical assessment for people living with mental disorders in the U.S. [34]. This reflects a growing appreciation of how a person’s ethnic culture shapes their experience and understanding of their mental health condition [35]. There is increasing awareness in mental health research and clinical practice of the connection between one’s ethnic identity and their resources for coping, social support, and resilience [36].

### 1.2. Personal Recovery and Psychological Well-Being

Although there is no single definition of recovery for those who are living with chronic mental health problems, personal recovery is a concept that refers to individualized processes of improving one’s well-being and working toward a meaningful life, whether or not symptoms are present [37,38]. In the narratives of people with mental illness, personal recovery has been described as developing new attitudes, goals, or skills, or regaining a positive sense of self, beyond the effect mental illness has had on their lives [39]. Ralph [40] described the dimensions of mental health recovery in terms of internal factors, such as an individual’s capabilities and coping; external factors, like connections to important activities and people; and empowerment, involving self-determination in one’s treatment and overall quality of life. Personal recovery diverges from a functional or medical understanding of recovery (i.e., the absence of symptoms and impairment). Instead of a focus on a cure, conceptualizations of personal recovery can refer to enhancing one’s quality of life through hope and optimism, social connectedness, positive identity, resistance to stigma, and acceptance of one’s condition [17,41].

Psychological well-being, sometimes described as flourishing [42,43], is a useful construct for examining the connection between ethnic identity development and a young adult’s recovery. Psychological well-being and personal recovery are distinct, conceptually related constructs that share a number of common components. Psychological well-being includes theoretical dimensions such as positive self-image and social relationships, and a sense of autonomy, external mastery, purpose in life, and personal growth [44]. These factors have been identified in structural models of subjective recovery [40] and integrated by frameworks and measures of personal recovery processes [17,45]. This conceptual overlap occurs because personal recovery can involve the unique processes by which individuals pursue their own notions of psychological well-being. In other words, the concept of personal recovery essentially affirms that psychological well-being can exist in the midst of living with a mental illness. While there is limited research studying ethnic identity in personal recovery processes, ethnic identity development has been positively associated with psychological well-being in numerous cross-sectional and longitudinal studies of young adults from minoritized ethnic and racial groups in the U.S. [11,46,47,48]. Examining how young adults experience a connection between their ethnic identities and psychological well-being can elaborate on the relationship between ethnic identity development and personal recovery that we observed in our initial quantitative results [24]. The present study’s analyses focus on two questions drawn from these concepts to enhance the interpretation of our results. (1) How is ethnic identity related to participants’ psychological well-being and recovery experiences? (2) How do descriptions of recovery vary between participants with higher and lower levels of ethnic identity development?

## 2. Materials and Methods

### 2.1. Research Design

This study is the second component of an explanatory sequential mixed methods design [49]. It builds upon and expands our first quantitative study of ethnic identity in young adults living with mental disorders [24]. An explanatory sequential design involves collecting and analyzing quantitative data in the first component, then using the results from the quantitative study to determine the research questions and analytic plan for a second, qualitative study component. When that qualitative study is completed, the two types of data are integrated to generate additional findings [23]. This study design was selected to gain insight into the quantitative data through qualitative and mixed methods [49].

### 2.2. Participants

Participants were enrolled in a randomized trial testing a mental health service engagement program for young adults [50]. The trial was conducted with two large, urban, publicly funded behavioral health organizations that provide recovery-oriented services to adults in the Eastern U.S. Participants were eligible for the trial if they were (a) aged 18–34; (b) living with a mental illness; (c) enrolled in a psychiatric rehabilitation program conducting the trial; and (d) a recipient of social welfare services that indicate increased social vulnerability (e.g., government health insurance, nutrition assistance, juvenile justice or child welfare systems). A sample of young adults living with mental illness was recruited across four sites between February 2018 and February 2020.

### 2.3. Measures

The quantitative measures assessed self-reported personal characteristics and social history. Participants’ health records were used to assess their primary psychiatric diagnoses, which were then grouped into diagnostic categories [34]. Ethnicity and race were categorized into three groups that corresponded with the participant self-reports: Hispanic/Latine (i.e., Latin American origins), Black (non-Hispanic/Latine), and multiracial (reporting more than one group). Current symptoms were measured by the Colorado Symptom Index (CSI; [51]). The 12-item, 4-point Multigroup Ethnic Identity Measure (MEIM; [26]) scale was used as a global assessment of ethnic identity, consisting of affirmation and pride in one’s ethnic group, together with a sense of belonging and engaging in behaviors that indicate involvement with one’s ethnic group. Example items include “I am happy that I am a member of the group I belong to” and “I participate in cultural practices of my own group, such as special food, music, or customs”. Personal mental health recovery was measured using the Recovery Assessment Scale (RAS; [45]). The RAS is frequently used for self-rating mental health recovery within five factors: personal confidence and hope, willingness to ask for help, goal and success orientation, reliance on others (i.e., social supports), and no domination by symptoms. The RAS items use a 5-point Likert scale for how strongly participants agree or disagree with statements like “I’m hopeful about my future” and “coping with my mental illness is no longer the main focus of my life”.

### 2.4. Procedure

Prospective participants were informed of the research by staff at their rehabilitation programs and invited to contact the research team if they were interested in joining the study. Further details on recruitment and the randomized trial are described in the published study protocol [50]. Interviews started with the survey measure items and ended with the qualitative questionnaire. The qualitative interview guide was designed to elicit participants’ mental health narratives, with a focus on how they experienced and managed their mental health. Participants were also asked what their ethnic group membership means to them and how it has affected topics related to recovery. For example, “how do you think your ethnic identity has affected the way you get help with your mental health?” The qualitative questions have been used successfully in similar studies [52,53] and were effective in the pilot testing for the current study. Interviews were audio-recorded and lasted approximately 1 h. The research team conducted qualitative interviews with participants enrolled in the trial until we observed that continued data collection was not adding greater complexity or depth to the data [54]. This resulted in the qualitative interviews analyzed for the present study. All the participants gave their informed consent, and the study protocols were approved by the sponsoring university’s Committee on Activities Involving Human Subjects.

The research team was made up of men and women, aged 25–53, from White (i.e., European origins), Black, Asian, Hispanic/Latine, and multiracial backgrounds. All the team members had training in research interviewing and professional and/or personal experience related to living with a mental illness. The authors were all members of the research team and have advanced training in both providing services and conducting research in mental health care settings. The team addressed positionality related to age, race, ethnicity, socioeconomic status, training, and professional identity during the research process through intentional consideration and management of how our social positions and identities might impact the data collection and analyses.

### 2.5. Analytic Strategies

We used descriptive statistics to summarize participants’ characteristics and scores on the CSI, MEIM, and RAS scales. For the qualitative data, the audio-recorded interviews were professionally transcribed. The analysts used a directed content analysis approach that was both inductive and deductive [55] in order to add context and supporting (or non-supporting) evidence to our quantitative results. To start, two authors read all the interview transcripts and identified participants’ expressions of psychological well-being [44,56], as well as statements related to ethnic identity [11,12] and the personal recovery factors measured by the RAS [45], in the quantitative data. ATLAS.ti (Version 24.1.1) [57] was used for open coding and to develop a list of initial codes and operational definitions for each code based on the research questions and concepts informing the study [58]. Next, three authors independently coded a subset of the interview transcripts using the initial codes and met to refine and reach a consensus on the coding frame. We decided which coded quotations were representative of the predetermined codes, and data relevant to the research questions that could not be coded with a predetermined code were labelled with emergent codes. All the transcripts were then recoded and organized into categories and salient themes based on the final hierarchical coding frame. This process resulted in a codebook that included themes and subthemes, descriptions, and exemplar participant quotes.

To address the second research question, the participant interviews were divided into two groups, according to whether their ethnic identity scores were above or below 2.50, the midpoint for the MEIM (low = 1.00–2.50; high = 2.51–4.00). The grouped data were examined using joint displays [59] to compare the codes and coded quotations between groups. The methodological rigor and integrity of the analyses were strengthened by an audit trail of process and analytic memos, and periodic debriefing with the research team to ensure that analyses were accurately answering the research questions.

## 3. Results

### 3.1. Participants

The participant characteristics for the current study are described in Table 1. Participants ranged in age from 19 to 29 years old. All described themselves as being part of minoritized Hispanic/Latine or Black ethnic groups. Among the Hispanic/Latine participants, the ethnic groups included Dominican, Puerto Rican, Mexican, and Ecuadorian. Among those who were not Hispanic/Latine, the specific ethnic groups named by participants were African-American, Nigerian, and West Indian (e.g., origins in Jamaica, Haiti, the Bahamas). The multiracial participants all described themselves as part of the groups above through at least one parent, and most also had one parent who was White. The ethnic identity mean score for the sample was within the range (2.94–3.21) reported in previous studies of young adults from the same ethnic and racial groups [24]. The majority of participants were males and had the schizophrenia spectrum (i.e., psychosis) as their primary diagnoses. Most reported mental illness symptomology above the clinical threshold (CSI ≥ 30 [51]), indicating that the frequency and severity of their symptoms were similar to other adults diagnosed with a chronic mental illness. Almost all had been hospitalized for their symptoms in the past. Nearly, a third of participants had experienced homelessness and only three were employed at the time of the study.

### 3.2. Qualitative Findings

Participants made both positive and negative connections between their psychological well-being and recovery, and aspects of their ethnic identities. Three overarching themes were identified as characterizing these aspects: (1) cultural history, traditions, and values; (2) mental illness stigma within the ethnic community; and (3) bias and discrimination in mental health services. These themes are presented below with the subthemes in italics and exemplar quotes from participant narratives.

#### 3.2.1. Cultural History, Traditions, and Values

Participants discussed how appreciating their ethnic group’s history and values, and taking part in its traditions, contributed to *overcoming challenges* they faced in their lives. They primarily referred to challenges related to the effects of mental illness and traumatic experiences, and some also mentioned other difficult life experiences up to the present. Participants shared insights into their ethnic group’s culture. Several expressed *pride* in their group, highlighting what they saw as their group’s positive characteristics. Some emphasized their group’s histories of perseverance in the face of economic hardship, immigration, or social injustices as supporting a sense of *empowerment* in their own lives.

*Being Black is very empowering for me, just because learning about the history of our people, all the social movements that we’ve started, and how far we’ve come just through all the fighting and all the nonsense that we’ve had in America.*

Many participants referred to their ethnic culture’s *social support* and *religious traditions* as important for their personal development and recovery. They described their ethnic communities as interdependent, intergenerational social networks, usually encompassing immediate and extended family. They viewed their communities as significant sources of emotional, material, and informational support. One participant illustrated the essential role that his ethnic culture plays in managing his mental health:

*How I was brought up in more of a traditional way with the community here, the traditions and routines that my grandmother taught us, I mean, it plays a big role in my life. If I wasn’t taught the way I was taught, I will have nothing to fall back on in times of trouble or in times of need. I think the structure taught me how to get over things.*

Speaking about the salience of religious beliefs and practices in his ethnic community, he expressed how they are helpful for maintaining his *optimism* in terms of recovery:

*It’s “go to church or pray on these issues” and it coincides with the medication. I know I have the spiritual willingness to do something, to become something better, and I have people praying for me and motivating me.*

Some participants felt their ethnic cultures were supportive of *seeking help* for mental health, but they also discussed difficulties with relying on their ethnic community for recovery support. Cultural preferences for getting help within one’s own ethnic community could present barriers to receiving other kinds of professional treatment that participants said they benefitted from. A participant who identified as Dominican and Ecuadorian explained this through contrasting his experiences with each community’s approach to dealing with mental health problems:

*Dominicans, they like to give themselves their own advice. My Ecuadorian side, you know, is different. They wanted me to go out and get [other kinds of] help. They’re very humble people…it rubs off on you. I learned from them to never think you know everything.*

Participants also expressed that recovery processes could depend significantly on the quality of attachment in familial relationships. Not being in regular, meaningful contact with one’s family was perceived as a serious vulnerability for people in their ethnic communities. One participant gave the following example:

*We confide in our own if we can. So, if you don’t have any strong ties, sometimes people will turn to drugs or abusing medication. It’s a really big issue if people don’t have a support system.*

#### 3.2.2. Mental Illness Stigma within the Ethnic Community

Participants also perceived high levels of stigma within their ethnic communities around mental illness and utilizing professional mental health treatment. They articulated a need for more culturally appropriate mental health information and services for people from their ethnic groups. They felt that having limited information led to *negative stereotypes* of people with mental illness (e.g., “*dangerous*”, “*weak or incapable*”). Some also perceived doubts among ethnic community members about whether mental illness was a valid health problem, rather than a moral or spiritual problem. They also described how concerns about stigma toward people with mental illness and their families contributed to shame and concealment. One participant explained that negative attitudes about treatment were deeply rooted in the fear of public stigma for people within her ethnic community:

*There is a belittling of people with mental health issues. It’s assumed that they can’t carry out their daily lives. I definitely believe it’s because of stigma and lack of knowledge that people just dismiss you if you are said to have a mental health condition. If you know that they’ll write you off, it’s very hard to have that discussion. It has to be normalized within our community. And I feel like once that discussion is normalized, then getting treatment can be talked about.*

Participants discussed how they resisted stigma within their ethnic communities. Many described developing *autonomy* in their mental healthcare, and in other areas of their lives, as important for being persistent in their recovery. One participant gave an example of needing to think and act more independently in order to cope with negative attitudes in his community about needing mental healthcare:

*I love my culture. It’s a blessed culture. But it’s tough. I’m not going to lie. Because certain people, they be like, “oh, you don’t need that kind of help”. They be like, “no necesito eso. No me gusta eso de retar las cosas así. I don’t need that stuff”. But I know who I am, I know what I’m about, so I’m not going to listen to that stuff.*

#### 3.2.3. Bias and Discrimination within Mental Health Services

Participants expressed intersectional burdens related to being from a minoritized ethnic–racial group and living with a mental illness. They conveyed how accessing mental health services could be challenging and stressful due to *societal discrimination* and *interpersonal biases.* Several stated that professional mental health services were viewed with *mistrust* in their communities. They recalled witnessing skepticism among family and community members about mental health professionals and the treatments they recommended (e.g., therapy, medication, rehabilitation services). Participants thought this was due to limited English language fluency or lower acculturation, and to being less familiar with conventional U.S. mental health services.

Participants also connected mistrust with perceived racism. They highlighted conditions of structural and institutional racism that were shaping experiences with mental healthcare for people in their ethnic groups. Participants identified socioeconomic disadvantages that they believed were affecting their group’s access to treatment. They also noted a lack of linguistically and culturally appropriate treatment options. Commenting on what he saw as the obstacles faced by Black Americans, one participant described compounded hurdles to accessing treatment:

*Some understand that they need the help, but they don’t know how to get it. And they have to deal with money, the cost of insurance, of transportation, the location, how far the distance is to travel. And I guess sometimes we basically can’t find a place or voice to be able to express it in a positive way.*

In addition, participants described interpersonal bias among mental health professionals and felt it was a common experience for people from their ethnic groups. Recounting how she faced excessive hurdles and racist attitudes when trying to find high-quality care, one participant remarked:

*My community is met with those stereotypes attached to them. It’s like, “oh, you don’t deserve services because you somehow are the sole cause of your problems. You aren’t someone to be helped”. It’s this weird idea that we don’t deserve the help that we’re seeking.*

She described a lengthy ordeal, experiencing symptoms while trying to locate and contact affordable mental health professionals in her area, and waiting months for appointments. She ultimately found acceptable treatment, noting:

*the way that I dealt with it was finding a therapist that had the same cultural background, but that is not at all easy to find, so there are a lot of barriers.*

### 3.3. Mixed Methods Results

The integrated findings are presented in Table 2. The frequencies for participant narratives that contained personal recovery factors [45] were determined for the total sample (*n* = 44) and for both the high (*n* = 28) and low (*n* = 16) ethnic identity groups. More than half of participants (63%) were in the high ethnic identity group. This aligns with the exploratory analyses in the previous quantitative study component, where ethnic identity was positively associated with personal recovery in the larger sample of 83 young adults (b = 0.71, 95% CI [0.24, 1.24]) [24]. Nearly all the participants discussed their experiences of seeking help and receiving support from others in their recovery. However, all the participants who referred to help-seeking barriers related to stigma in their ethnic communities, a lack of appropriate services for their ethnic group, or racial bias and discrimination were in the high ethnic identity group (*n* = 20). Mental health professionals were the primary sources of recovery support discussed by both the high and low ethnic identity participants. The majority of participants in the high ethnic identity group also cited family as a source of recovery support (*n* = 19; 79.1%), compared to notably fewer (*n* = 6; 46.1%) in the low ethnic identity group. To a lesser degree, both groups also spoke about receiving support from friends and other informal sources.

Over two-thirds of the sample expressed optimism and positive views of themselves. Slightly more than half of participants spoke about feeling better and being able to cope more effectively with their mental health conditions. There were no notable differences in the content of their narratives. Less than half of participants discussed their aspirations or spoke about pursuing goals in life. Only participants in the high ethnic identity group discussed negative stereotypes in their ethnic communities about people with mental illness having limited capabilities or potential in life.

## 4. Discussion

This study demonstrates how ethnic identity promotes mental health recovery for minoritized young adults through providing social support and enhancing their psychological well-being and resilience. The findings complement and extend our initial quantitative results by presenting context for the associations between the study participants’ personal recovery and their pride, attachment, and involvement in their ethnic groups. Overall, our mixed methods study contributes to understanding the aspects of ethnic identity development that are relevant to recovery for Black and Hispanic/Latine young adults living with mental illness in the U.S. and offers insight into experiences of intersectional stigma and structural racism that interfere with recovery among this marginalized population.

The connection between participants’ ethnic identities and personal recovery was most evident in their descriptions of the social support they gained from their ethnic communities. Their involvement with religious and extended familial communities was a significant source of social connectedness and offered participants’ people who they could rely on for support and hope in their recovery processes. Our findings are aligned with prior research showing that belonging to a cultural or religious community, or using spiritual healing practices, is a prominent theme in the personal recovery narratives of racial and ethnic minority populations [16,17]. Our young adult sample were part of the Black and Hispanic/Latine ethnic groups in the U.S. who tend to prioritize communalism [61]. Participants underscored how attachment and interpersonal relationships played a critical role in the cultural characteristics of social support in their groups. They noted that family were often the ones who motivated them to seek help and persist in their recovery, and most participants who said they received support from family members in their recovery had high scores on the ethnic identity measure [26]. This reflects the strong collectivistic family orientation, referred to as *familismo*, observed within Hispanic/Latine cultures [29] and appears consistent with research indicating that normative cultural expectations of responsibility to the family and community have protective effects on the well-being of Hispanic/Latine young adults [28]. Our findings also echo research showing how Black Americans cope with adversity through communalism and spiritual practices derived from African cultural traditions [61]. The church, in particular, is a trusted institution among Black Americans that promotes communalism, racial and ethnic pride, and social support [30,61].

The positive connections participants described between ethnic identity and psychological well-being suggest that a developed sense of ethnic identity is a competency or an asset that may facilitate processes of connectedness, hope, positive identity, and empowerment in personal recovery. These findings support our initial quantitative study [24], as well as research showing associations between ethnic identity development and psychological well-being in minoritized young adults [11,46,47,48]. Participants’ narratives demonstrated that they had considerable insight into their ethnic identity group’s culture and traditions, and they made several associations between their own ethnic identities and psychological well-being. Their knowledge and expressions of positive feelings about their ethnic group, along with their reflections on how belonging to their ethnic group has affected their lives, are distinct indications of healthy ethnic identity development [11,27]. This corresponds to ethnic identity scores above the midpoint for the study sample mean (M = 3.07; SD = 0.52) and for more than half of individuals in the sample. Participants also described their ethnic identities as promoting psychological well-being through positive relationships (i.e., social support) and as internalized positive characteristics of their group (e.g., pride, optimism/religious faith, empowerment). Akin to the notion of collective or social consciousness [62], ethnic identity provides a community with shared norms and beliefs that validated their senses of self. These attributes are consistent with conceptualizations of personal recovery and processes that are often identified in the narratives of people living with mental illness [16,17].

However, the relationship between ethnic identity development and personal recovery was not exclusively positive for participants. Our qualitative findings add important nuance to these processes for Black and Hispanic/Latine young adults. Participants identified characteristics of their ethnic culture, along with intersectional stigma and discrimination, that could get in the way of their recovery. Their perceptions are consistent with studies that show mental illness stigma, limited mental health literacy, and concerns about whether their cultural needs will be met are commonly reported barriers to seeking and participating in mental healthcare for young adults from minoritized ethnic and racial groups [52,53,63]. Some participants felt that insular aspects of their ethnic identity groups presented obstacles to receiving needed professional treatment. So, while communalism confers certain benefits, it may also have drawbacks for recovery. This has been reflected in transcultural psychiatry research that describes how cultural schemas influence the perceived relevance and value of professional treatments [35] and determine which approaches to healing and recovery are preferred by an ethnic community [36]. Participants also emphasized the influence of public stigma around mental illness in their communities. Research on personal recovery narratives has found that in collectivist cultures with limited psychiatric literacy, mental illness stigma often adversely affects both the individual and their family [16]. Mental illness stigma related to perceptions of one’s family members can be especially detrimental to help-seeking among collectivistic minority groups. This is because concerns around mental illness discrimination against a family member may be intensified among groups that already experience significant racial discrimination in healthcare and in society [64].

Our findings also illustrate the inequitable developmental challenges faced by ethnically–racially minoritized young adults in recovery. Participants with more developed ethnic identities spoke more about, and seemed to be more sensitized to, experiences of stigma and discrimination, as well as conditions of structural racism. Ethnic identity development processes that involve gaining knowledge about the history and traditions of one’s group often increase awareness of racism, which may heighten the harmful effects that discrimination has on psychological well-being [29]. In addition to reaching the common milestones of adulthood (e.g., pursuit of education and occupational goals, adult life roles), research on psychosocial development indicates young people must contemplate their heritage and position as members of their ethnic group in order to construct a healthy self-concept [11,12]. Young people from minoritized groups are required to understand themselves in relation to both their own group and the majority group, and they must also reckon with bias and discrimination directed at them for being minorities [11,12]. In studies of personal recovery, individuals from minoritized racial and ethnic groups have described themselves as also recovering from the detrimental effects of intersecting racism and mental illness stigma [17,65]. They describe processes of recovery as requiring both internal and external resources to overcome the interpersonal and societal disadvantages they face [65]. Structural barriers (e.g., limited access and affordability) are known to prevent minoritized young adults from receiving mental health treatment [7,9,63]. Research suggests services that are responsive to both cultural identity and societal inequalities are needed to support recovery among minoritized young adults living with mental illness [22,66].

We also found that minoritized young adults with developed ethnic identities had considerable resilience against discrimination. Being able to access treatment and support for their recovery while they were exposed to racism and experiencing mental illness demonstrated that study participants were able to resist stigma and make effective use of the resources available to them. This is affirmed in research on ethnic identity development showing that adaptive coping skills, such as viewing marginalization as a function of external forces instead of inherent characteristics, are often cultivated through being sensitized to racism early in life (e.g., racial socialization) [30,61] and that these competencies are ultimately protective for ethnically and racially minoritized people throughout adulthood [11,15,29]. However, participants’ experiences also demonstrate that stigma and structural racism can generally reduce opportunities to develop self-determination in mental health recovery. Racial and ethnic discrimination in healthcare placed constraints on participants’ access to treatment, which can limit their power to choose from a variety of interventions and recovery strategies. Public stigma likely limited their exposure to positive images of people from their own ethnic communities who have lived experience of mental illness. Some participants were able to exercise autonomy in resisting stigma. Although it represented a positive health behavior, increased separation or independence from the community may attenuate the protective influences conferred by their ethnic group’s traditional cultural practices, religious values and familial networks (i.e., collective consciousness). Participants’ experiences illustrate how mental healthcare options that require assimilation into the American majority group and/or resisting discrimination are inequitable and are likely to preserve mental health disparities for people from minoritized groups. This suggests that substantial improvements to mental healthcare access and services, including culturally appropriate options for care, are needed to facilitate recovery for minoritized young adults.

Our findings suggest that approaches to care in which clinicians are attentive to ethnic identity-related barriers, and to strengths that can be drawn upon in treatment [30], may be particularly relevant for minoritized young adults in recovery-oriented mental health services. To strengthen ethnic identity and psychological well-being, mental health practitioners could engage young adults in conversations about the positive aspects of their ethnic background, explore their community’s heritage and accomplishments, or assist with increasing their participation in traditional cultural practices [30]. Research also shows that stress related to experiencing racism may require clinical attention [67]. There are some evidence-supported techniques clinicians can employ that may help alleviate symptoms and improve coping (see the *Healing Racial Trauma protocol* [30] for examples). Culturally responsive interventions that consider a young adult’s developmental stage, along with the cultural and social context, have shown promise for improving outcomes for minoritized young adults living with mental disorders [68]. Evidence supports mental health interventions adapted to incorporate cultural components (e.g., language, metaphors, values, traditions) that are important for youth from specific minoritized ethnic and racial groups [69]. Recent research also supports person-centered practices that are based on assessment of the cultural context and engaging patients to articulate which aspects of their identity [70] and social environment [71] matter most for their mental health. Instead of tailoring interventions for specific groups, individual patients’ perspectives on their cultural and social context can inform case formulations and guide clinical care. Our findings also indicate that the scope of clinical practice needs to include approaches based on critical theories (e.g., intersectionality, critical race theory) [72] and structural competency [73]. Interventions to ameliorate the burdens of intersectional discrimination are required to address societal factors that act as barriers to mental health recovery. Empirical research on how to implement these approaches in clinical practice is limited, but clinicians can acquire knowledge and skills to advocate for young adults who are socially marginalized [74]. A standard of care that involves clinicians intervening with local, regional, and global institutions and advocating for policies to improve access to mental health services on behalf of minoritized patients, or collaborating with minoritized communities to address structural vulnerabilities for young adults, should be a goal for mental health professionals [75].

## 5. Conclusions

This study identified salient connections between ethnic identity development and personal recovery from the perspectives of Black and Hispanic/Latine young adults living with mental illness in the U.S. The findings suggest ethnic identity could play an important role in supporting recovery and reducing mental health disparities among racially and ethnically minoritized young adults. This is one of few studies to examine ethnic identity in personal mental health recovery and to explain how ethnic identity processes can promote social support, hope, positive self-image, and empowerment. The findings provide insights into future clinical and research approaches for this understudied field. While our study was limited to the experiences of Hispanic/Latine, Black, and multiracial young adults, studies show that young adults living with mental illness from Asian and Native American groups in the U.S. also experience risks of health disparities and barriers to recovery [76]. Subsequent studies should also include the experiences of these young people. Research is still needed on how to integrate cultural assets and ethnic identity development into recovery care for minoritized young adults and on the development and testing of mental health interventions that mitigate intersectional discrimination. Offering culturally appropriate interventions in recovery-oriented clinical services may help more minoritized young adults with mental illness to experience well-being and lead a meaningful life.

## Figures and Tables

**Table 1:** Participant characteristics, *n* = 44.

		*n* (%)	M(SD)
Age in Years			26.74 (3.33)
Gender			
	Male	28 (63.6%)	
	Female	16 (36.4%)	
Ethnicity and Race			
	Hispanic/Latine	18 (40.9%)	
	Black, Non-Hispanic/Latine	15 (34.1%)	
	Multiracial	11 (25.0%)	
Diagnosis			
	Schizophrenia Spectrum	25 (56.8%)	
	Depressive	15 (34.1%)	
	Bipolar	4 (9.1%)	
Current Symptoms ^1^			39.84 (7.73)
History of Hospitalization		39 (88.6%)	
History of Homelessness		13 (29.5%)	
Secondary Education		34 (77.2%)	
Employed		3 (6.8%)	
Ethnic Identity ^2^			3.07 (0.52)

^1^ Colorado Symptom Index [60]; ^2^ Multigroup Ethnic Identity Measure [26].

**Table 2:** Differences in participant narratives by content and ethnic identity score, *n* (%).

Code ^1^ and Description	Total	High EI ^2^	Low EI ^2^	Content Difference
Willingness to ask for help: statements about seeking help for mental illness and related needs	39 (88.8%)	25 (89.2%)	14 (87.5%)	High EI group described stigma and racial discrimination as help-seeking barriers.
Reliance on others: statements about receiving support from others to recover from mental illness	37 (84.0%)	24 (85.7%)	13 (81.2%)	Fewer in low EI group cited family as a source of support.
Personal confidence, hope: positive statements about oneself and the future	30 (68.1%)	22 (78.5%)	8 (50.0%)	None.
No domination by symptoms: statements that suggest psychiatric symptoms are interfering less with life	25 (56.8%)	17 (60.7%)	8 (50.0%)	None.
Goal and success orientation: statements about having or pursuing personal goals	20 (45.4%)	14 (50.0%)	6 (37.5%)	High EI group described negative stereotypes about people with mental illness.

^1^ Personal recovery factors; ^2^ Ethnic identity.

## Data Availability

The data presented in this study are available on request from the corresponding author due to privacy restrictions.
